# Cell-specific conditional deletion of interleukin-1 (IL-1) ligands and its receptors: a new toolbox to study the role of IL-1 in health and disease

**DOI:** 10.1007/s00109-020-01928-5

**Published:** 2020-05-29

**Authors:** Emmanuel Pinteaux, Wesam H Abdulaal, Ilgiz A Mufazalov, Neil E Humphreys, Maj Simonsen-Jackson, Sheila Francis, Werner Müller, Ari Waisman

**Affiliations:** 1 Faculty of Biology, Medicine and Health, University of Manchester, AV Hill Building, Oxford Road, Manchester, M13 9PT United Kingdom; 2 Biochemistry Department, Faculty of Sciences, King Abdulaziz University, P.O.BOX 80203, Jeddah, 21589 Kingdom of Saudi Arabia; 3 Institute for Molecular Medicine, University Medical Center of the Johannes Gutenberg-University of Mainz, Langenbeckstrasse 1, Building 308A, 55131 Mainz, Germany; 4 Epigenetics and Neurobiology Unit, Adriano Buzzati-Traverso Campus, EMBL-Rome, Via Ramarini, 3200015 Monterotondo, RM Italy; 5 Department of Infection, Immunity & Cardiovascular Disease, Medical School, University of Sheffield, S10 2RX, Sheffield, United Kingdom

**Keywords:** Inflammation, Immunity, IL-1, IL-1 receptors, Cre/loxP, Conditional deletion

## Abstract

The pro-inflammatory cytokine interleukin-1 (IL-1) plays a key role in many physiological processes and during the inflammatory and immune response to most common diseases. IL-1 exists as two agonists, IL-1α and IL-1β that bind to the only signaling IL-1 type 1 receptor (IL-1R1), while a second decoy IL-1 type 2 receptor (IL-1R2) binds both forms of IL-1 without inducing cell signaling. The field of immunology and inflammation research has, over the past 35 years, unraveled many mechanisms of IL-1 actions, through in vitro manipulation of the IL-1 system or by using genetically engineered mouse models that lack either member of the IL-1 family in ubiquitous constitutive manner. However, the limitation of global mouse knockout technology has significantly hampered our understanding of the precise mechanisms of IL-1 actions in animal models of disease. Here we report and review the recent generation of new conditional mouse mutants in which exons of *Il1a*, *Il1b*, *Il1r1*, and *Il1r2* genes flanked by loxP sites (^fl/fl^) can be deleted in cell-/tissue-specific constitutive or inducible manner by Cre recombinase expression. Hence, IL-1α^fl/fl^, IL-1β^fl/fl^, IL-1R1^fl/fl^, and IL-1R2^fl/fl^ mice constitute a new toolbox that will provide a step change in our understanding of the cell-specific role of IL-1 and its receptor in health and disease and the potential development of targeted IL-1 therapies.

## Introduction

Interleukin-1 is a master pro-inflammatory cytokine implicated in a wide range of physiological processes including development [1], regulation of neuroimmune and neuroendocrine functions [2], and central processes such as sleep and memory [3] and plays a key role in the initiation and orchestration of the inflammatory response to most, if not all, pathological inflammatory conditions, including infections and non-communicable diseases such as atherosclerosis or stroke (see [4] for review). The IL-1 family comprises two IL-1 agonists (IL-1α and IL-1β) and the naturally IL-1 receptor antagonist (IL-1Ra) that bind primarily to the IL-1 type 1 receptor (IL-1R1) and IL-1 receptor accessory protein (IL-1RAcP) leading to cell signaling (reviewed in [5]), while a second IL-1 type 2 receptor (IL-1R2) binds both IL-1 isoforms without inducing cell signaling, acting therefore as a decoy receptor [6].

Since their discovery, IL-1α and IL-1β are believed to have similar and often overlapping biological functions, since they bind to the same receptor inducing similar cell signaling mechanisms. However, there are marked differences in the regulation of expression and mechanisms of actions of these two cytokines. Although both isoforms require enzymatic cleavage for generation of their mature forms, IL-1β is the main secreted isoform, whereas IL-1α remains cytoplasmic but can also be released during cell death or by mechanisms that are different from that of IL-1β (reviewed in [7]). Furthermore, both IL-1α and IL-1β have been thought to exert similar biological activities primarily through binding to IL-1R1. However, several previous studies have demonstrated differential actions of both cytokines in various paradigms of inflammation; for instance, IL-1α and IL-1β exert differential potency at inducing fever when administered exogenously [8], while IL-1α, but not IL-1β, triggers sepsis lethality in mouse [9] and is required for T cell activation during allergen-induced hypersensitivity [10]. Further, IL-1α, but not IL-1β, induces brain cells to generate the LG3 neuroprotective protein fragment of the extracellular matrix component perlecan, a prominent component of the blood-brain barrier [11]. Of interest, polymorphisms in the human *Il1a*, but not *Il1b* gene, is associated with higher incidence of vascular malformation and/or higher risk of ischemic stroke [12, 13]. In contrast, IL-1β, but not IL-1α, activates IL-6 expression in neurons [14], selectively mediates the response to vascular injury [15], while IL-1α- and IL-1β-specific actions have also been identified in acute colon inflammation in mice [16]. Taken together, these observations suggest that IL-1α and IL-1β may be differentially expressed during inflammation and may exert non-overlapping ligand-specific differential actions dependent on the disease paradigm.

## Mouse genetic models to understand the role of IL-1α and IL-1β in disease

For decades, the field of inflammation research has unraveled key mechanisms of IL-1 actions using traditional global gene targeting knockout technology in animal models. Indeed, IL-1α-deficient (^−/−^), IL-1β^−/−^, and IL-1α/β^−/−^ (as well as IL-1Ra^−/−^) mice generated by Horai and collaborators in 1998 [17] have proven useful to identify some selective mechanisms of actions of both isoforms in some pathological conditions. In those genetic models, disruption of the *Il1a* and *Il1b* genes was achieved by deletion of the NH_2_-terminal coding region for mature IL-1α (exon 5–intron 5) and IL-1β (exon 3–5), leading to ubiquitous constitutive inhibition of expression of either genes. These genetic models have been used widely in many disease models and have subsequently led to the identification of some IL-1α- and IL-1β-specific mechanisms as described above. Further, IL-1R1^−/−^ mice, originally generated by Immunex by targeted deletion of exon 1 and 2 of the *Il1r1* gene [18], showed that most, but not all, IL-1 actions are mediated by IL-1R1 (see [19] for review). Indeed, studies using IL-1R1^−/−^ mice in animal models of gut infection with helminth *Trichuris muris* [20] and experimental stroke [21] found that IL-1β can function in an IL-1R1-independent manner, while IL-1β exacerbates neuronal apoptosis caused by status epilepticus through a mechanism independent of IL-1R1 [22]. Further, some neuroprotective actions of IL-1 are believed to be triggered independently of IL-1R1 via activation of the neuroprotective PI3K/Akt signaling pathway [23], while we have reported IL-1R1-independent IL-1 actions in glial cells [24]. Those IL-1R1-independent actions, primarily observed in the original IL-1R1^−/−^ mice, are known to be mediated through a spliced variant of the *Il1r1* gene leading to a truncated IL-1R1 isoform still expressed upon exon 1–2 deletion, due to the activation of an additional internal promoter positioned upstream of exon 1–2 [25]. This truncated isoform of the receptor has been fully characterized and lacks part of the extracellular IL-1 binding region but is still capable of inducing an intracellular signal in response to IL-1 that is known to mediate the neuroprotective actions of IL-1 in the brain via activation of the PI3K/Akt pathways [25]. Ubiquitous *Il1rap* gene deletion, that encodes IL-1RAcP, has also been achieved in mice, by targeting exon D1 and part of exon D2 that encode the first Ig-like and part of the second Ig-like extracellular domains, resulting in complete inhibition of IL-1 signaling in response to IL-1α and IL-1β [26]. In accordance with the phenotypic responses observed in IL-1R1^−/−^ mice, IL-1RAcP^−/−^ mice show reduced neuroimmune and febrile responses to IL-1 [27, 28]. Finally, IL-1R2^−/−^ mice in which exon 2–4 are deleted using conventional gene targeting method have also been generated [29]. These mice show increased susceptibility to collagen-induced arthritis, while IL-1β-induced cytokine response was enhanced in macrophages. In agreement with its inhibitory function, IL-1Ra^−/−^ mice develop spontaneous autoimmune arthritis [30] and psoriasis-like cutaneous inflammation [31] and show increased brain injury to experimental stroke [32] and atherosclerotic lesion in experimental atherosclerosis [33]. Taken together, these observations demonstrate the complexity of the IL-1 system and point to important, yet undiscovered, mechanisms of actions of IL-1 ligands and their receptors, which cannot be explored by using classical pharmacological or genetic approaches.

## Generation of a new toolbox to allow cell-specific conditional deletion of IL-1 ligands and their receptors

**Fig. 1:**
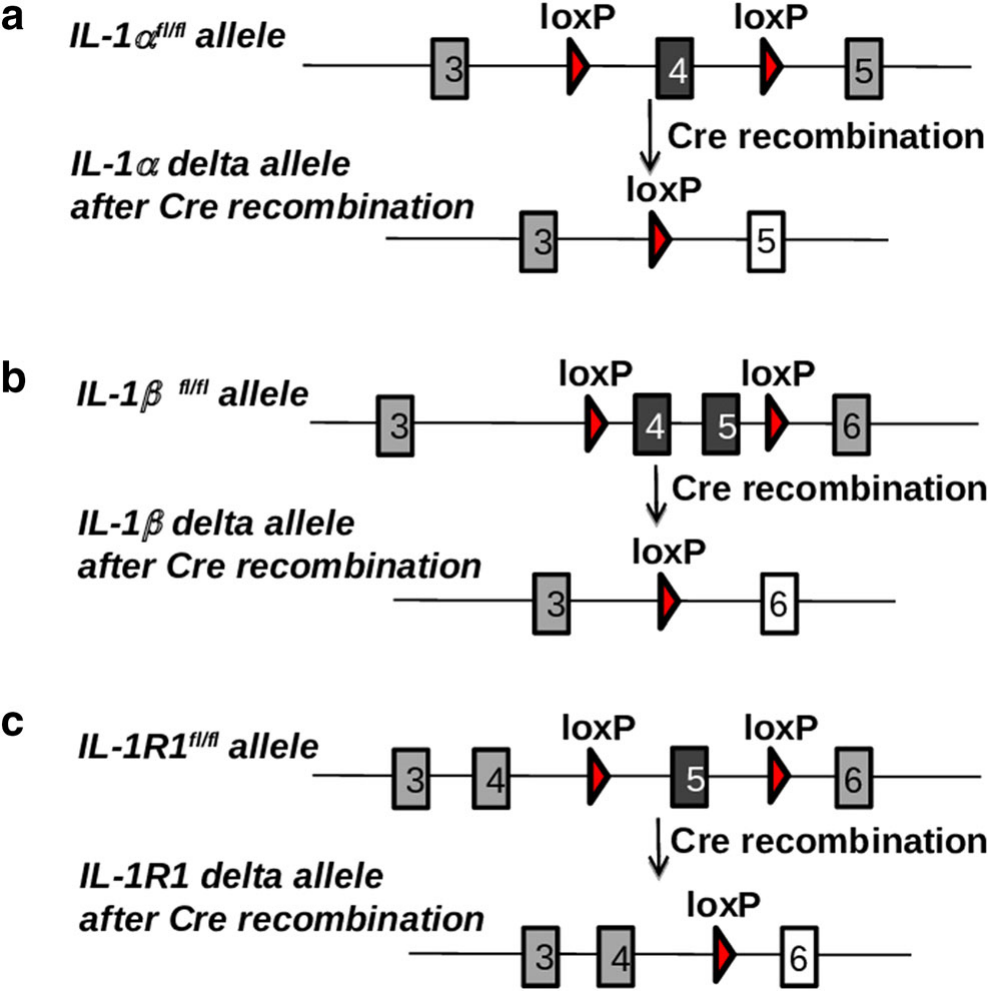
Generation of IL-1α^fl/fl^, IL-1β^fl/fl^, and IL-1R1^fl/fl^ mice. **A** Exon 4 of the *Il1a* gene (for IL-1α ^fl/fl^), **B** exon 4–5 of the *Il1b* gene (for IL-1β ^fl/fl^ mice), or **C** exon 5 of the *Il1r1* gene (for IL-1R1^fl/fl^) flanked with loxP sites is excised upon Cre recombination, resulting in cell-specific IL-1α-, IL-1β-, or IL-1R1-deficient allele, respectively

Germline gene deletion in mice has yielded important discoveries regarding the role of IL-1 ligands and their receptors in various inflammatory paradigms. However, this approach has important limitations such as viability and fertility of progeny, subtle phenotypic changes, and/or compensatory mechanisms that may alter steady-state immune responses. In relation to the IL-1 system, IL-1α^−/−^, IL-1β^−/−^, IL-1R1^−/−^, IL-1RAcP^−/−^, and IL-1R2^−/−^ mice have all been reported to be viable with no obvious altered fertility. However, some reports suggested that IL-1 regulates ovulation, oocyte maturation, and early embryonic development [34, 35], which could lead to long-term significant phenotypic changes. Indeed, normal bone growth and remodeling are altered in IL-1R1^−/−^ and IL-1α/β^−/−^ mice [36, 37], whereas decrease body fat mass is reduced in IL-1Ra^−/−^ mice [38], strongly suggesting that significant phenotypic changes occur after ubiquitous deletion of IL-1 family members. Finally, significant compensatory changes are known to occur after ubiquitous gene deletion, and microarray analysis demonstrated that expression of several genes is altered in IL-1R1^−/−^ mice [24]. To overcome these limitations, the Cre/loxP system that allows selective/conditional deletion of targeted genes was recently used to generate new mouse mutant lines to allow cell-specific conditional deletion of IL-1 ligands and its receptors in a Cre recombinase-dependent manner (loxP-flanked, abbreviated as fl/fl). To this end, we have recently reported the generation and characterization of new IL-1α^fl/fl^ and IL-1β^fl/fl^ mouse lines [39, 40] generated from Il1a^tm1a(EUCOMM)Wtsi^ (clone EPD0822-4-H02) or Il1b^tm1a(EUCOMM)Hmgu^ (clone HEPD0840-8-E03) embryonic stem cells purchased from the European Mouse Mutant Cell Repository (EuMMCR). The full description of the gene targeting strategies for both IL-1α^fl/fl^ and IL-1β^fl/fl^ mice as well as experimental procedure from initial culturing and microinjection of ES cells leading to the generation of mice allowing for the conditional deletion of IL-1α and IL-β are published [39, 40]. In these new alleles, exon 4 of the *Il1a* gene (for IL-1α^fl/fl^ mice) or exon 4–5 of the *Il1b* gene (for IL-1β^fl/fl^ mice) flanked with loxP sites can be deleted by Cre recombinase, leading to exon 4 or 4–5 deletion and generation of cell-specific IL-1α and IL-1β-deficient allele, respectively (Fig. 1A and B).

**Fig. 2:**
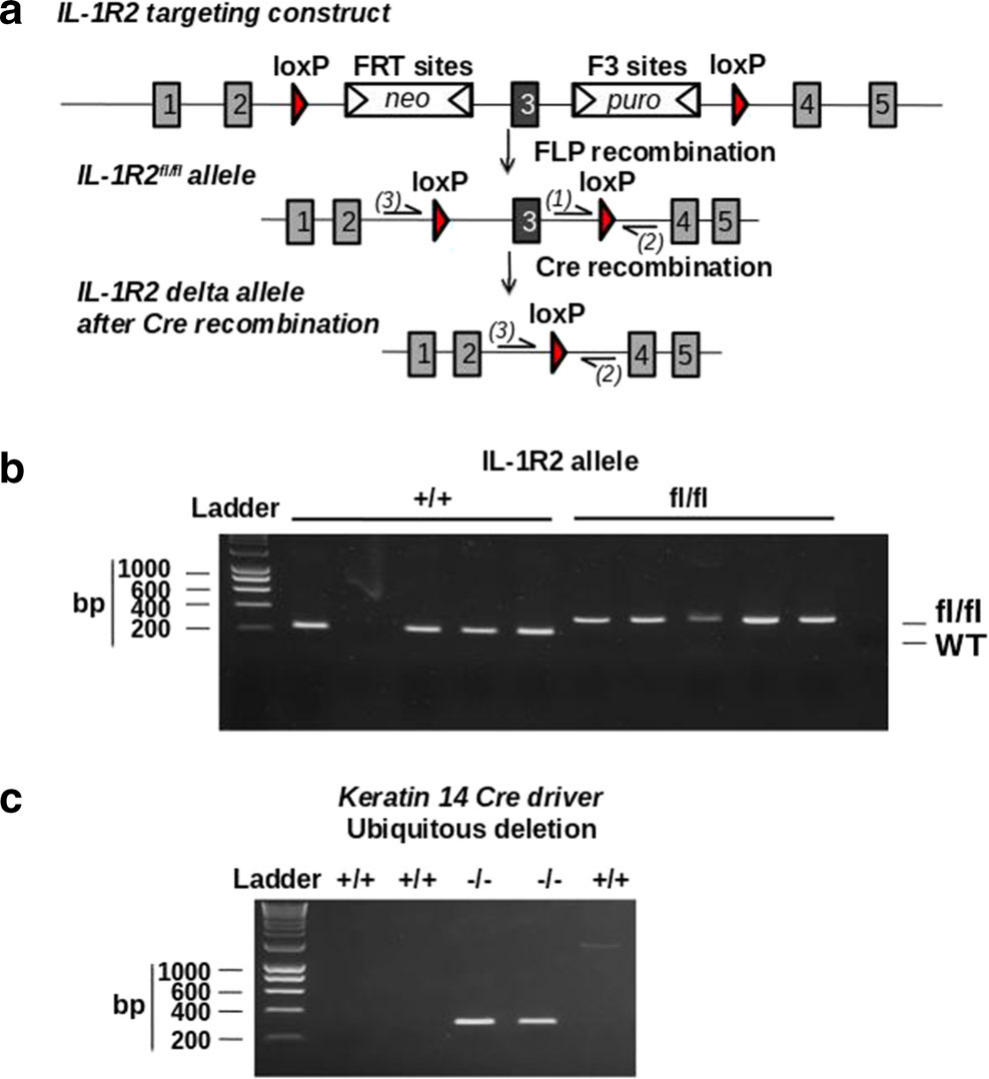
Generation of IL-1R2^fl/fl^ and IL-1R2^−/−^ mice. **A** Genetic approach to generate IL-1R2^fl/fl^ mice was designed to induce deletion of exon 3 encoding part of the extracellular binding domain, generating a frameshift from exon 4 to all downstream exons leading to genetic inhibition of IL-1R2. **B** Genotyping identification of IL-1R2^fl/fl^ mice was carried out by PCR using the following primers: Forward, TGTCTCCATCAGACTGACTTTAGG, depicted *(1)*, and reverse, ACCATGTCTGCCTGTTCACC, depicted *(2)* on genomic DNA. Amplification product size obtained was as follows: wild type (228 bp) and IL-1R2^fl/fl^ (347 bp). **C** Genotypic identification of exon 3 deletion in IL-1R2^−/−^ mice (obtained by crossing IL-1R2^fl/fl^ mice with mice expressing Cre recombinase under a keratin 14 promoter) was carried out by PCR on isolated genomic DNA using the following primers: Forward, GTAGTGGGCAATCAGATGGAC, depicted *(3)*, and reverse, ACCATGTCTGCCTGTTCACC, depicted *(2)*. Amplification product size obtained was 300 bp in the IL-1R2−/− mice after Cre recombination

Recently, two new mouse mutants, allowing for the conditional deletion of *Il1r1* (IL-1R1^fl/fl^), have been described. Robson and collaborators [41] have generated a new IL-1R1^fl/fl^, in which exon 3–4 of the *Il1r1* gene (encoding part of the extracellular binding region) can be deleted by Cre recombination and demonstrated ubiquitous inhibition of IL-1R1 signaling by the crossing of the conditional allele to the CMV-Cre mice, which mediated recombination in early embryogenesis. Concomitantly, we have generated a new IL-1R1^fl/fl^ mouse (developed by Taconic, Cologne, Germany), in which exon 5 that also encodes part of the extracellular binding region of the receptor is flanked by LoxP sites [42] (Fig. 1C). In those two new IL-1R1 mutants, deletion of exon 3–4 or exon 5 inactivates the two previously described functional IL-1R1 gene transcripts (including the full-length IL-1R1 and truncated IL-1R3) upon Cre- mediated recombination [25]. In our study, we have also reported the generation of a new ubiquitous IL-1R1^−/−^ mouse as well as myeloid cell-specific IL-1R1-deficient mice by crossing IL-1R1^fl/fl^ with mice expressing Cre recombinase under the promoter of keratin 14 (K14-Cre) and the Vav promoter, respectively [42]. Of importance, an advanced genetic tool based on restoration of *Il1r1* gene expression has been developed by Liu and collaborators [43]. In this advanced model, excision of a disruptive intronic sequence in the *Il1r1* gene under Cre recombination in a global IL-1R1^−/−^ background allows functional restoration of IL-1 signaling under cell-specific promoters and has been important in the discovery of mechanisms of IL-1 signaling in the brain in the broad context IL-1-driven central inflammation [44]. Finally, generation of IL-1R2^fl/fl^ targeting exon 3 of the *Il1r2* gene was also reported [45], and we now report in our hand the generation of a similar IL-1R2^fl/fl^ mouse that targets exon 3 of *Il1r2* gene and further generation of IL-1R2^−/−^ by crossing IL-1R2^fl/fl^ with mice expressing Cre recombinase under the promoter of keratin 14 (K14-Cre) (Fig. 2A).

## Generation of IL-1R2^fl/fl^ and IL-1R2^−/−^ mice

IL-1R2 conditional (IL-1R2^fl/fl^) mice were generated at Taconic (Cologne, Germany) by gene targeting using BAC clones as the targeting vector from the C57BL/6J RPCI-23 BAC library encoding two loxP sites flanked exon 3 of the *Il1r2* gene and subsequent homologous recombination in C57BL/6 N embryonic stem (ES) cells. From targeted C57BL/6 N ES cells, as verified by southern blotting, chimeric mice were generated and bred to C57BL/6 females. Germline transmission was identified by genotyping PCR sample analysis using a Caliper LabChip GX device (details are available upon request). Genotyping identification of IL-1R2^fl/fl^ mice was carried out on genomic DNA by PCR (see Fig. 2B) (details of primers used in Fig. 2 legend and protocol of DNA amplification available upon request). Amplification product size obtained were as follows: wild type (228 bp) and IL-1R2^fl/fl^ (347 bp).

A new ubiquitous IL-1R2^−/−^ mouse was generated by crossing the IL-1R2^fl/fl^ mice with mice expressing Cre recombinase under the control of the human keratin 14 promoter in oocytes as described [46], leading to genetic deletion of exon 3 in all tissues. The deletion of exon 3 causes a frame shift from exon 4 to all downstream exons. Genotypic identification of exon 3 deletion in IL-1R2^−/−^ mice was carried out by PCR on isolated genomic DNA (see Fig. 2C) (details of primers used in figure legend and protocol of DNA amplification available upon request).

## Advanced discoveries in mechanisms of IL-1 actions in disease using the toolbox

The new toolbox comprising IL-1α^fl/fl^, IL-1β^fl/fl^, IL-1R1^fl/fl^, and IL-1R2^fl/fl^ mice allows for the first time the generation of new mouse lines in which IL-1 and its receptors can be deleted in a cell/tissue specific manner. To date, IL-1α^fl/fl^, IL-1β^fl/fl^, IL-1R1^fl/fl^, and IL-1R2^fl/fl^ mice have been crossed with specific Cre drivers leading to cell- or tissue-specific deletion of either gene in a constitutive (Cre) or inducible (Cre-ER) manner, revealing new mechanisms of IL-1 actions. Table 1 provides a list of the cell-/tissue-specific deficient mice in IL-1α, IL-1β, IL-1R1, and IL-1R2 generated to date that have been tested under different inflammatory paradigms. While the generation of cell-specific IL-1α and IL-1β-deficient lines is limited, due to the recent generation of IL-1α^fl/fl^ and IL-1β^fl/fl^ mice, the first studies using those models have demonstrated the critical role of microglial IL-1β in the establishment of pain in the context of complex regional pain syndrome [39], while cardiomyocyte-derived IL-1α has been found not to contribute to tissue remodeling during myocardial infarction [40]. In contrast, IL-1R1^fl/fl^ mice have generated various cell-/tissue-specific IL-1R1-deficient lines, most studies showing a critical role for IL-1 signaling in immune cell activation and vascular activation in various models of peripheral infection and chronic inflammation (see Table 1). For instance, IL-1 signaling in cells of the hematopoietic lineage is required for the IL-17 and IL-22 response to gut infection by the nematode *Trichuris Muris* [42], whereas IL-1 signaling in T cells [50] and in GM-CSF producing cells [52] plays a critical role in experimental autoimmune encephalomyelitis. Furthermore, IL-1 signaling in T cells plays a key role in the systemic immune response to injection of CD3 antibodies [49]. Inhibition of IL-1 signaling by ColVI-Cre driver in intestinal mesenchymal cells showed that IL-1R1 in these cells has no important role in the development of intestinal carcinogenesis [60]. Furthermore, no direct role of IL-1 signaling in CD45^+^ hematopoietic was found in IL-1-mediated resistance to *Mycobacterium tuberculosis* [62]. In contrast, IL-1R1 on hepatocytes reduces liver injury in a model of acute liver failure [61], while deletion of IL-1R1 in pancreatic cells alters glucose homeostasis and triggers β-cell de-differentiation [57]. Finally, a study using Ly6G-Cre mice found that IL-1R1 in neutrophils plays a key role in reducing the tumorigenic effects of IL-1 [51]. In peripheral vascular beds, cadherin-Cre mediated IL-1R1 deletion in endothelial cells contributes to the anti-tumor function of adoptively transferred T cells regulated by IL-1β [63], whereas IL-1R1 signaling in smooth muscle cells contributes to the atheroprotective effect of IL-1 in advanced atherosclerotic lesions [56].

**Table 1:** List of cell-/organ-specific deficient mice for IL-1 isoforms or their receptors and main effects observed

Gene	Cre driver	Cell/tissue targeted	Main effects	References
Il1a	Myh6-Cre	Cardiomyocytes	No effect on cardiac tissue remodeling after MI	Bageghni et al. [40]
Il1b	CMV-Cre	Ubiquitous deletion	Reduces bone metastasis during breast cancer	Tulotta et al. [47]
	CX3CR1-CreER	Microglial cell	Reduces the development of pain	[39]
Il1r1	K14-Cre	Ubiquitous deletion	Mediates peripheral immune response to *T. Muris* infection	[42]
			Inhibits melanoma inflammatory niche	Young et al. [48]
	CMV-Cre	Ubiquitous deletion	Decreases inflammatory responses to systemic challenges	Mufazalov et al. [49]; Mufazalov et al. [50]
			NI	Robson et al. [41]
	PGK-Cre	Ubiquitous deletion	Regulates cardiac tissue remodeling after MI	Bageghni et al. [40]
	Col1a2-CreER	Fibroblasts	Regulates cardiac tissue remodeling after MI	Bageghni et al. [40]
	CD4-Cre	T cells	Regulates immune response to CD3 antibody injection	Mufazalov et al. [49]
			Regulates neuroinflammation in EAE	Mufazalov et al. [50]
	Vav-Cre	Myeloid cells	Mediates peripheral immune response to T. Muris infection	Adbulaal et al. [42]
	Ly6G-Cre	Neutrophils	Reduces the tumorigenic effect of IL-1	Dmitrieva-Posocco et al. [51]
	Csf2-Cre	GM-CSF positive cells	Regulates inflammation after EAE	Komuczki et al. [52]
	CX3CR1-CreER	Microglial cells	Reduces renewal of microglial population	Bruttger et al. [53]
			Regulates microglial activation after CNS inflammation	Zhu et al. [54]*
			No effect on febrile response to IL-1	Knoll et al. [55]*
	Myh11-CreER	Smooth muscle cells	Reduces atheroprotective effect of IL-1 after atherosclerosis	Gomez et al. [56]
	Pdx1-Cre	Pancreatic cells	Alters glucose homeostasis and triggers β-cell de-differentiation	Burke et al. [57]
	Slco1c1-CreER	Brain endothelial cells	Reduces CNS inflammation and brain damage after stroke	Wong et al. [58]
			Reduces fever response to IL-1	Matsuwaki et al. [59]
	Nestin-Cre	Neuronal cells	No effect on febrile response to IL-1	Matsuwaki et al. [59]
			Reduces brain damage after stroke	Wong et al. [58]
	Trpv1-Cre	Nociceptor sensory neurons	No effect on febrile response to IL-1	Matsuwaki et al. [59]
	htPA-Cre	Neural crest cells	No effect on febrile response to IL-1	Matsuwaki et al. [59]
	ChAT-Cre	Catecholaminergic neurons	Decreases brain damage after stroke	Wong et al. [58]
	PF4-Cre	Platelets	No effect on brain damage after stroke	Wong et al. [58]
	ColVI-CreER	Intestinal mesenchymal cells	No effect on development of intestinal cancer	Koliaraki et al. [60]
	Hep-Cre	Hepatocytes	Reduces liver injury after acute liver failure	Gehrke et al. [61]
	CD45-Cre	Leukocytes	No role in IL-1-mediated resistance to *Mycobacterium tuberculosis*	Bohrer et al. [62]
	Cdh5-Cre	Vascular endothelial cells	Mediates the anti-tumor properties of T cells. Regulates IL-1-induced brain inflammation	Lee et al. [63]
	Tie2-Cre	Endothelial cells	Decreases IL-1-induced brain inflammation	Liu et al. [44]^#^
Il1r2	CMV-Cre	Ubiquitous deletion	Reduces inflammation after arthritis	Martin et al. [45]
	K14-Cre	Ubiquitous deletion	NI	Unpublished**

For IL-1R1^fl/fl^ mice, all cell-/tissue-specific IL-1R1^−/−^ mice have been generated by IL-1R1^fl/fl^ mouse from Abdulaal et al. [42], except those marked (*), generated by IL-1R1^fl/fl^ mouse from Robson et al. [41]. #In the study of Liu and collaborators (2019), the following mouse lines have also been generated: IL-1R1^fl/fl^ x LysM-Cre, IL-1R1^fl/fl^ x CX3CR1-Cre, IL-1R1^fl/fl^ x Camk2a-Cre, IL-1R1^fl/fl^ x Vglut2-Cre, and IL-1R1^fl/fl^ x GFAP-Cre. **IL-1R2^fl/fl^ mice crossed with K14-Cre mice are reported in the present publication. *Cre*, constitutive deletion by Cre drivers; *Cre-ER*, inducible deletion by Cre-ER drivers; *EAE*, experimental autoimmune encephalomyelitis; *MI*, myocardial infarction; *NI*, not indicated

In the brain, IL-1 signaling in microglia is required for the renewal properties of microglial progenitor cells [53]. Brain endothelial IL-1R1 is essential in the initiation of the fever response elicited by IL-1, whereas deletion of IL-1R1 on central or peripheral neurons (including catecholaminergic neurons and nociceptor sensory neurons) had no noticeable effect on the febrile response [59]. An interesting work by Knoll and collaborators [55] confirmed that endothelial IL-1R1 signaling is critical in the establishment of the febrile response to IL-1, whereas IL-1R1 signaling in microglia of the brain parenchyma has no role. Further, endothelial IL-1R1 is essential for endothelial activation in the context of IL-1-driven brain inflammation [44] and a further study using microglial-specific IL-1R1-deficient mice showed that IL-1 actions on brain endothelia triggers the production of endothelial-derived factors that are able to activate microglial cells [54]. In the context of stroke, brain endothelial IL-1R1, but also neuronal IL-1R1, is critical in mechanisms of cerebrovascular inflammation and brain damage after experimental cerebral ischemia, whereas no involvement of IL-1 signaling in peripheral cells, including platelets on stroke outcome was observed [58].

Finally, little work has been conducted regarding IL-1R2^fl/fl^ mice, and to date, only ubiquitous constitutive deletion of IL-1R2 (IL-1R2^−/−^) has been achieved, including that of our work. The only work reporting the use of IL-1R2^−/−^ in disease is that of Martin and collaborators [45], who demonstrated that IL-1R2 deletion plays an important inhibitory role on IL-1-regulated inflammation in a model of arthritis, in accordance with its known inhibitory function, as recently reviewed [64].

## Concluding remarks and future directions

IL-1 is a key cytokine regulating many physiological processes as well as the inflammatory responses to infection or injury, and global constitutive IL-1-deficient mouse models, which is a fairly recent approach, have to date helped unraveling key mechanisms of IL-1 actions in disease but have significant limitations. The recent generation of new mouse mutants allowing conditional deletion of IL-1 and its receptors has led to the discovery of unexpected new mechanisms of inflammation regulated by IL-1. To date, a limited number of cell-/tissue-specific IL-1-deficient mice have been generated, and this is mainly due to the fact that IL-1α^fl/fl^, IL-1β^fl/fl^, IL-1R1^fl/fl^, and IL-1R2^fl/fl^ mice have only been recently produced. However, several projects targeting the IL-1 system in other cells/tissues are currently ongoing. Importantly, to the best of our knowledge, IL-1Ra^fl/fl^ and IL-1RAcP^fl/fl^ mice have not been generated yet, and future generation of new lines in which all IL-1 ligands and their receptors can be targeted in other cell/tissue and other disease models will lead to new mechanisms to be discovered, providing a step change in our understanding of IL-1 actions disease and the potential development of new targeted IL-1 therapies.
